# Commentary: Power-washing the brain with the heart-lung machine?

**DOI:** 10.1016/j.xjon.2020.08.013

**Published:** 2020-08-29

**Authors:** Gianni D. Angelini, Tomas A. Salerno

**Affiliations:** a British Heart Foundation, Bristol Heart Institute, Bristol University, Bristol, United Kingdom; b Division of Cardiothoracic Surgery, University of Miami Miller School and Jackson Memorial Hospital, Miami, Fla

**Figure:**
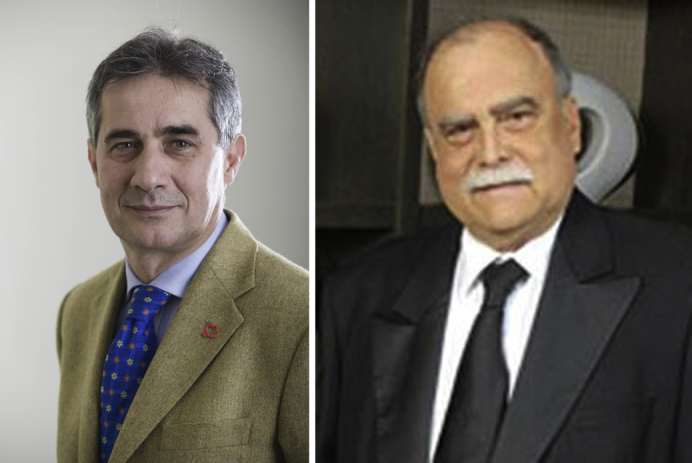
Gianni D. Angelini, MD, and Tomas A. Salerno, MD

**Central Message**

Neurologic injury may occur during coronary bypass surgery and may be accentuated by the use of a heart-lung machine. Continued efforts are needed to understand and minimize this injury.

See Article page 1.

In this issue of the *Journal*, Browne and colleagues^1^ report the incidence and describe the importance of covert stroke during coronary artery bypass surgery (CABG) in 49 patients who had diffusion-weighed magnetic resonance imaging (DW-MRI) of the brain, showing 39% with perioperative covert stroke, 6% with clinical stroke, 26% with delirium, and 10% with no stroke. These are important findings for such a routine procedure. Here we provide some insight into this problem.

### Brain Injury After Cardiac Surgery

Brain injury is a major complication of cardiac surgery and significantly increases the likelihood of the need for long-term care. Perioperative stroke occurs in 2%-6% of all patients. More than 20% of patients aged >65 years and 33% of those aged >80 years experience postoperative delirium. The rate of postoperative cognitive dysfunction is estimated to exceed 80% at discharge, and the dysfunction persists in 25% of patients at 1 year. Brain injury also may trigger chronic or progressive dementia.^2^, ^3^, ^4^

### Mechanism of Brain Injury in Cardiac Surgery

It is assumed that brain injury is triggered by release of microemboli (microscopic atherosclerotic particles and/or air bubbles) in the bloodstream, which are carried to the brain. Indeed, intraoperative transcranial Doppler (TCD) monitoring demonstrates showers of small particulate matter and/or air emboli during CABG.^5^ Abnormal fluorescein angiography, suggestive of retinal microvascular damage, has been reported in patients undergoing CABG with cardiopulmonary bypass, and “particles” in the blood (gas or microemboli) in the cerebral circulation, detected with transcranial Doppler ultrasound, have been observed more often than during off-pump CABG.^5^

The relationship between intraoperative brain embolic load and brain injury remains to be clarified, however. Some studies have reported that the embolic burden detected by TCD monitoring is associated with early cognitive deficits, whereas others have not confirmed this finding.^3^^,^^6^

### MRI to Detect Perioperative Brain Injury

MRI examination of the brain is the “gold standard” for identifying and quantifying perioperative brain injury and has been used widely in randomized controlled trials investigating neuroprotective interventions in cardiac surgery.^7^, ^8^, ^9^ Various imaging techniques are used to identify markers of such injury, including structural and functional MRI and DW-MRI. Abu-Omar and colleagues^9^ used functional MRI to show that patients undergoing on-pump CABG, but not those undergoing off-pump CABG, have a significant relative reduction in prefrontal activation, which correlates with intraoperative cerebral microembolic load. The main advantage of DW-MRI is that DW-detectable lesions typically appear within 2 hours of surgery and represent “new” injury, so a baseline scan is not needed to confirm that the lesion was not present before surgery. Another advantage is that the new framework for defining stroke proposed by the American Heart Association/American Stroke Association,^7^ includes neuroimaging (together with clinical and pathological evidence), and thus lesions found on DW-MRI count as “silent” brain injury, even in the absence of obvious clinical findings.

DW-MRI lesions following left heart valve surgery are reported in approximately 50% of patients.^10^^,^^11^ These lesions are multiple and very small, ranging from 1 to 10 mm in diameter and from 32 to 750 mm^3^ in volume. They are located in all cerebrovascular territories but most frequently in frontal and watershed border zones, and the pattern of distribution confirms an embolic basis. Few (∼9%) are associated with overt clinical signs of stroke, and they represent “silent” brain injury in most cases.^10^^,^^11^

### Clinical Relevance of Silent Brain Injury

In population-based studies, a strong association exists between silent brain injury identified by MRI and prevalent cognitive dysfunction and dementia.^12^^,^^13^ Therefore, it is plausible that a similar relationship exists between appearance of new lesions after cardiac surgery and neurocognitive decline. Some preliminary data have suggested that the appearance of new silent brain lesions after CABG is associated with early postoperative neurocognitive deterioration.^14^ Further investigations with longer follow-up are needed.

The use of postoperative cognitive dysfunction as a marker of perioperative brain injury is problematic because of potential difficulties in ascertainment. Multiple factors affect neurocognitive test performance during the first week after surgery, particularly treatment of postoperative pain, sedation, and other clinical recovery issues. Many, if not most, patients experience some degree of cognitive dysfunction in the immediate postoperative period. Such a nearly universal occurrence is clearly not an appropriate marker of brain injury. Only after this period has passed can objective assessment of the patient's cognition be performed, although the duration of altered cognition after surgery, required to define postoperative neurocognitive decline, has not been clearly defined.^10^

Cardiac surgery is increasingly being offered to older, higher-risk patients with comorbidities, and thus the incidence of neurologic complications is likely to increase in the future. The report from Browne and colleagues adds another piece to the puzzle of our understanding and knowledge of brain injury after heart surgery.
